# *Public Health Nutrition* special issue on ultra-processed foods

**DOI:** 10.1017/S1368980017002853

**Published:** 2018-01

**Authors:** Bridget Kelly, Enrique Jacoby

**Affiliations:** 1 Early Start, School of Health & Society University of Wollongong Northfields Avenue Wollongong, NSW 2522, Australia; 2 Global Health Advocacy Incubator, Brazil Coordinator Rua Capote Valente 500, Apt 220, São Paulo 05409000, Brazil; 3 Former Regional Advisor on Nutrition of PAHO/WHO

This special issue on ultra-processed foods considers the role and utility of food processing-based classification systems in food and nutrition research and public policy. Such classification systems have been developed as industrialised food processing and the transnational corporations that drive the production, advertising and sales of industrially processed foods^(^ ^1^ ^)^ have become a major influencer in the global food system and an important determinant of nutrition-related health outcomes^(^ ^2^ ^)^.

As highlighted by Monteiro and colleagues in this special issue^(^ ^2^ ^)^, in the past decade, food processing-based classification systems have been increasingly acknowledged in reports and commentary from the WHO, FAO and the Pan American Health Organization (PAHO). During this time, such systems have also been applied in nutrition monitoring and in epidemiological and intervention studies published in *Public Health Nutrition* and other journals.

Globally, there are at least seven frameworks that have been devised for classifying foods according to their level of food processing^(^ ^3^ ^,^ ^4^ ^)^. The most commonly applied framework is the NOVA system, first defined in a commentary published in *Public Health Nutrition* in 2009^(^ ^5^ ^)^ and further refined to include four food groups: unprocessed or minimally processed foods, processed culinary ingredients, processed foods and ultra-processed foods^(^ ^2^ ^)^. Originally developed in Brazil, its global application is evident from the range of countries represented in this special issue, spanning Latin America, North America, Europe, the Middle East and Australia. The term ‘ultra-processed’ foods is unique to the NOVA system and is defined as ‘…not modified foods but formulations made mostly or entirely from substances derived from foods and additives, with little if any intact food’^(^ ^2^ ^)^.

Ultra-processed foods dominate the food supply in high-income countries and this is increasingly the case in low- and middle-income countries, where urbanisation, a globalised industrial food supply including massively increased foreign direct investment in locally based food companies^(^ ^6^ ^)^, and mass and now social media marketing have dramatically shifted population diets away from unprocessed or minimally processed foods and freshly prepared meals^(^ ^7^ ^)^. Population nutrition monitoring studies presented in this special issue highlight this dominance of ultra-processed foods, particularly in North America and Europe but also in Latin America. According to these studies, all applying the NOVA system to national 24 h dietary recall data, the energy contribution of ultra-processed foods was 57·6 % for children and adults in the USA^(^ ^8^ ^)^, 35·9 % for French adults^(^ ^9^ ^)^, 29·8 % for Mexican children and adults^(^ ^10^ ^)^ and 28·6 % for Chilean children and adults^(^ ^11^ ^)^. It has been noted that ultra-processed foods contribute substantially to population intakes of micronutrients across many countries^(^ ^12^ ^)^. Findings from the studies in this special issue challenge that assertion and demonstrate an association between higher consumption of ultra-processed foods and poorer nutritional intakes, including higher intakes of energy and free/added sugar^(^ ^9^ ^,^ ^11^ ^)^ and lower intakes of fibre^(^ ^9^ ^)^, micronutrients^(^ ^9^ ^)^ and protein^(^ ^8^ ^)^.

Epidemiological evidence has previously demonstrated that ultra-processed food consumption is associated with poorer diet quality in the USA^(^ ^13^ ^)^, Canada^(^ ^14^ ^)^ and Brazil^(^ ^15^ ^)^; obesity in Brazil^(^ ^16^ ^)^, Guatemala^(^ ^17^ ^)^, Spain^(^ ^18^ ^)^ and Sweden^(^ ^19^ ^)^; hypertension in Spain^(^ ^20^ ^)^; metabolic syndrome in Brazil^(^ ^21^ ^)^; and dyslipidaemia in children in Brazil^(^ ^22^ ^)^. Papers in this special issue reinforce the aetiology between ultra-processed food consumption and nutritional outcomes and chronic conditions, and extend such evidence to new populations. Notably, two papers describe the relationship between ultra-processed food consumption and nutrition and health outcomes in First Nations peoples in Canada and are the first studies to apply NOVA to Indigenous populations. Higher energy contribution from ultra-processed foods was significantly associated with poorer diet quality^(^ ^23^ ^)^ and with metabolic syndrome^(^ ^24^ ^)^ in First Nations peoples. Percentage energy intake from ultra-processed foods has been proposed as a summary indicator of population diet quality^(^ ^25^ ^)^. Evidence presented in this special issue supports this idea, with ultra-processed food consumption being a better predictor of metabolic syndrome in an Indigenous Cree population compared with other indices of diet quality^(^ ^23^ ^)^. Other papers in this special issue find positive associations between ultra-processed food consumption and poorer diet quality (in Brazil^(^ ^26^ ^)^ and Colombia^(^ ^27^ ^)^), obesity (in young people^(^ ^28^ ^)^; and in adults in Europe^(^ ^29^ ^)^) and metabolic syndrome (in Lebanon^(^ ^30^ ^)^). To date, a small number of longitudinal studies have specifically applied the NOVA system for classifying diets and they support identified associations between higher ultra-processed food consumption and chronic conditions^(^ ^18^ ^,^ ^20^ ^,^ ^22^ ^)^. Other cohort studies (e.g.^(^ ^31^ ^)^) identify associations between specific ultra-processed products, such as sugary drinks, and nutritional and health outcomes. The reanalysis of existing cohort study data using the NOVA classification would be a worthy endeavour.

Food processing-based classification systems, and specifically NOVA, offer possibilities for use in public policy as a way to define unhealthful dietary patterns. This includes scope for use in ‘soft’ policies, including dietary advice, through to ‘hard’ policy options of laws, regulations and fiscal instruments. NOVA is used in the 2014 Dietary Guidelines for the Brazilian Population and the 2016 Dietary Guidelines for the Uruguayan Population, which both emphasise the consumption of unprocessed or minimally processed foods and recommend limiting processed foods and avoiding ultra-processed foods^(^ ^32^ ^,^ ^33^ ^)^. Two papers in this special issue examine the application of NOVA to the Brazilian dietary guidelines. These papers apply discourse and thematic analyses to explore the socio-ecological dimensions of nutrition that the new guide promulgates and (i) how this compares with the maiden version of the dietary guidelines from 2006^(^ ^34^ ^)^ and (ii) how this was received by stakeholders, as identified through submissions to the public consultation during its development^(^ ^35^ ^)^. Together these papers highlight the importance of Brazil’s legal framework and political mandate for adequate and healthy food as a human right in the Government’s avant-garde adoption of the NOVA system. More research is needed to understand consumers’ comprehension of, and their ability to adopt, dietary guidance based on levels of food processing. This is particularly relevant in some developed countries, where the loss of culinary skills has been documented^(^ ^36^ ^)^.

Food processing-based classification systems may underpin other regulatory strategies for the prevention and control of obesity and diet-related non-communicable diseases by identifying unhealthful foods. For example, NOVA has been applied in the Nutrient Profiling Model developed by PAHO to distinguish foods that require regulatory control^(^ ^37^ ^)^. Regulations may specify the restriction of ultra-processed foods from marketing to children, or prohibit nutrition and health claims from ultra-processed food products. Research in this special issue identifies that ultra-processed foods currently comprise almost all foods promoted during children’s television programmes in Argentina^(^ ^38^ ^)^ and that these products frequently carry nutrition and health claims in Australia^(^ ^39^ ^)^ and Canada^(^ ^40^ ^)^, which likely leads to health halo effects.

Food processing-based classification systems could also be applied in local planning regulations, where these seek to influence the availability or accessibility of foods in local environments. In a cross-sectional survey from one city in Brazil in this special issue, neighbourhood within-store availability of processed and ultra-processed foods was associated with children’s higher intakes of these foods and a lower intake of unprocessed foods^(^ ^41^ ^)^. In a separate Brazilian study, household food purchasing data identified a positive association between food purchasing at supermarkets and ultra-processed food intake, while ultra-processed food intake decreased with shopping at smaller markets and small producers^(^ ^42^ ^)^. These studies emphasise the need for local planning policies that restrict ultra-processed food availability and marketing in local food environments and for these to be context-specific.

Food policy interventions aimed at product reformulation typically aim to reduce at-risk or negative nutrients in processed and ultra-processed foods rather than shift consumption patterns towards less processed products. The commentary by Scrinis and Monteiro in this special issue highlights that reformulation to reduce negative nutrients can lead to ‘premium’ ultra-processed foods that are likely no healthier and often more expensive^(^ ^43^ ^)^. The challenging notion that food processing-based classification systems introduce is the need for a complete reorientation of global food supplies, away from ready-to-consume food and drink formulations and towards minimally processed foods and freshly prepared meals, rather than simply modifying ultra-processed foods to be less harmful. This notion is particularly complicated in countries where ultra-processed foods dominate food supplies and diets, and may pose an obstacle for the adoption of food processing-based classification systems in food policy. In these countries, such as the USA, UK and Australia, policies that seek to restrict the availability, accessibly, affordability or acceptability of ultra-processed foods would currently apply to the majority of market products and foods consumed. More research is needed to understand how such policies would be possible in such food environments and how consumers could adapt to a changing food supply in the context of poor culinary skills.

The evidence presented in this special issue confirms that food processing-based classification systems, and particularly NOVA, are useful in nutrition research designed to judge the quality of diets, dietary patterns, and components of food systems and environments. NOVA has already informed public policy specifications for the promotion of healthy diets, including healthy eating guidelines and the identification of foods requiring regulatory control. The apparent ability for this system to be adopted across countries and cultures suggests its potential for wider application in food policy.
